# Bromido[(1,2,5,6-η)-cyclo­octa-1,5-diene]methyl­platinum(II)

**DOI:** 10.1107/S1600536809052660

**Published:** 2009-12-12

**Authors:** Kwang Ha

**Affiliations:** a School of Applied Chemical Engineering, The Research Institute of Catalysis, Chonnam National University, Gwangju 500-757, Republic of Korea

## Abstract

In the title complex, [PtBr(CH_3_)(C_8_H_12_)], the Pt^II^ ion is in a distorted square-planar environment defined by the Br and methyl C atoms and the mid-points of the two π-coordinated double bonds of cyclo­octa-1,5-diene. As a result of the different *trans* influences of the Br atom and the methyl group, the Pt—C bonds *trans* to the methyl group [2.262 (11) and 2.261 (10) Å] are longer than those *trans* to the Br atom [2.118 (8) and 2.138 (9) Å].

## Related literature

For the crystal structure of [(cod)PtCl_2_] (cod = cyclo­octa-1,5-diene), see: Goel *et al.* (1982 ▶); Syed *et al.* (1984 ▶). For the crystal structures of [(cod)Pt(CH_3_)*L*] (*L* = OH, CH_3_ or Cl), see: Klein *et al.* (1999 ▶). For the crystal structure of [(cod)Pt(CH_3_)I], see: Nieger (2008 ▶). For related Pt–cot complexes, [(cot )Pt*X*_2_] (cot = cyclo­octa-1,3,5,7-tetra­ene; *X* = Br or I), see: Song *et al.* (2007*a* ▶,*b* ▶).
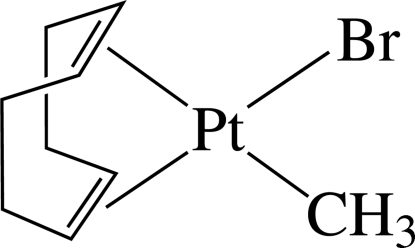


## Experimental

### 

#### Crystal data

[PtBr(CH_3_)(C_8_H_12_)]
*M* *_r_* = 398.21
Orthorhombic, 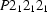

*a* = 7.1013 (15) Å
*b* = 11.184 (2) Å
*c* = 12.691 (3) Å
*V* = 1007.9 (4) Å^3^
*Z* = 4
Mo *K*α radiation
μ = 17.82 mm^−1^
*T* = 296 K
0.25 × 0.22 × 0.12 mm

#### Data collection

Bruker SMART 1000 CCD diffractometer
Absorption correction: multi-scan (*SADABS*; Bruker, 2007 ▶) *T*_min_ = 0.537, *T*_max_ = 1.000
7369 measured reflections
2514 independent reflections
1988 reflections with *I* > 2σ(*I*)
*R*_int_ = 0.041

#### Refinement

*R*[*F* ^2^ > 2σ(*F* ^2^)] = 0.032
*wR*(*F* ^2^) = 0.067
*S* = 1.04
2514 reflections
101 parameters
H-atom parameters constrained
Δρ_max_ = 0.92 e Å^−3^
Δρ_min_ = −1.27 e Å^−3^
Absolute structure: Flack (1983 ▶), 1023 Friedel pairs
Flack parameter: −0.02 (3)

### 

Data collection: *SMART* (Bruker, 2007 ▶); cell refinement: *SAINT* (Bruker, 2007 ▶); data reduction: *SAINT*; program(s) used to solve structure: *SHELXTL* (Sheldrick, 2008 ▶); program(s) used to refine structure: *SHELXTL*; molecular graphics: *ORTEP-3* (Farrugia, 1997 ▶); software used to prepare material for publication: *SHELXTL*.

## Supplementary Material

Crystal structure: contains datablocks global, I. DOI: 10.1107/S1600536809052660/nk2017sup1.cif

Structure factors: contains datablocks I. DOI: 10.1107/S1600536809052660/nk2017Isup2.hkl

Additional supplementary materials:  crystallographic information; 3D view; checkCIF report

## Figures and Tables

**Fig. 1.**
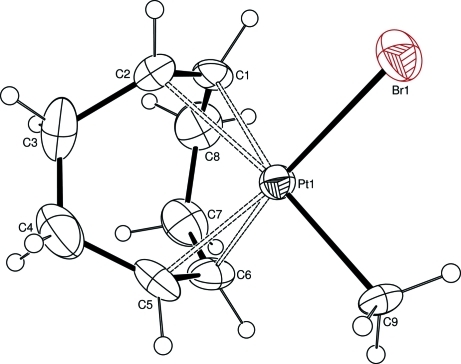
The structure of the title compound, with displacement ellipsoids drawn at the 30% probability level for non-H atoms.

## supplementary crystallographic information

### Comment

In the title complex, [PtBr(CH_3_)(C_8_H_12_)], the central Pt^II^ ion lies in a distorted square-planar environment defined by the Br and methyl C atoms and the two mid-points (M1, M2) of the π-coordinated double bonds of cycloocta-1,5-diene (cod) ligand (M1 and M2 denote the mid-points of the olefinic bonds C1—C2 and C5—C6, respectively) (Fig. 1). The Pt, Br, C9 atoms and the mid-points lie in a coordination plane with the largest deviation of 0.018 Å (M2) from the least-squares plane, and with bond angles in the range of 85.4°–94.5°. Because of the different *trans* influences of the Br atom and the methyl group, the Pt—C bonds *trans* to C9 of the methyl group (2.261 (10)–2.262 (11) Å) are longer than those *trans* to the Br atom (2.118 (8)–2.138 (9) Å). The cod ligand coordinates to the Pt atom in the twist-boat conformation with the coordinated double-bond lengths of 1.334 (13) and 1.367 (14) Å, and the cod ring angles lie in the range of 115.1 (10)°–127.3 (9)°.

### Experimental

To a solution of cyclooctadienedimethylplatinum(II) (0.1677 g, 0.503 mmol) in CH_2_Cl_2_/MeOH (15 ml/15 ml) was added acetyl bromide (0.0740 g, 0.602 mmol), and stirred for 5 h at room temperature. The solvent was removed under vacuum, the residue was washed with pentane and dried, to give a white powder (0.1611 g). Crystals suitable for X-ray analysis were obtained by slow evaporation from a methanol solution.

### Refinement

H atoms were positioned geometrically and allowed to ride on their respective parent atoms [C—H = 0.98 (CH), 0.97 (CH_2_) or 0.96 (CH_3_) Å and *U*_iso_(H) = 1.2*U*_eq_(C) or 1.5*U*_eq_(methyl C)]. The highest peak (0.92 e Å^-3^) and the deepest hole (-1.27 e Å^-3^) in the difference Fourier map are located 0.95 and 0.56 Å from the atoms Pt1 and Br1, respectively.

### Crystal data

**Table:**

[PtBr(CH_3_)(C_8_H_12_)]	*F*(000) = 728
*M**_r_* = 398.21	*D*_x_ = 2.624 Mg m^−^^3^
Orthorhombic, *P*2_1_2_1_2_1_	Mo *K*α radiation, λ = 0.71073 Å
Hall symbol: P 2ac 2ab	Cell parameters from 3683 reflections
*a* = 7.1013 (15) Å	θ = 2.4–28.4°
*b* = 11.184 (2) Å	µ = 17.82 mm^−^^1^
*c* = 12.691 (3) Å	*T* = 296 K
*V* = 1007.9 (4) Å^3^	Block, colourless
*Z* = 4	0.25 × 0.22 × 0.12 mm

### Data collection

**Table:**

Bruker SMART 1000 CCD diffractometer	2514 independent reflections
Radiation source: fine-focus sealed tube	1988 reflections with *I* > 2σ(*I*)
graphite	*R*_int_ = 0.041
ω scans	θ_max_ = 28.5°, θ_min_ = 2.4°
Absorption correction: multi-scan (*SADABS*; Bruker, 2007)	*h* = −8→9
*T*_min_ = 0.537, *T*_max_ = 1.000	*k* = −15→8
7369 measured reflections	*l* = −15→16

### Refinement

**Table:**

Refinement on *F*^2^	Secondary atom site location: difference Fourier map
Least-squares matrix: full	Hydrogen site location: inferred from neighbouring sites
*R*[*F*^2^ > 2σ(*F*^2^)] = 0.032	H-atom parameters constrained
*wR*(*F*^2^) = 0.067	*w* = 1/[σ^2^(*F*_o_^2^) + (0.0196*P*)^2^] where *P* = (*F*_o_^2^ + 2*F*_c_^2^)/3
*S* = 1.04	(Δ/σ)_max_ < 0.001
2514 reflections	Δρ_max_ = 0.92 e Å^−^^3^
101 parameters	Δρ_min_ = −1.27 e Å^−^^3^
0 restraints	Absolute structure: Flack (1983), 1023 Friedel pairs
Primary atom site location: structure-invariant direct methods	Flack parameter: −0.02 (3)

### Special details

**Table:**

Geometry. All e.s.d.'s (except the e.s.d. in the dihedral angle between two l.s. planes) are estimated using the full covariance matrix. The cell e.s.d.'s are taken into account individually in the estimation of e.s.d.'s in distances, angles and torsion angles; correlations between e.s.d.'s in cell parameters are only used when they are defined by crystal symmetry. An approximate (isotropic) treatment of cell e.s.d.'s is used for estimating e.s.d.'s involving l.s. planes.
Refinement. Refinement of *F*^2^ against ALL reflections. The weighted *R*-factor *wR* and goodness of fit *S* are based on *F*^2^, conventional *R*-factors *R* are based on *F*, with *F* set to zero for negative *F*^2^. The threshold expression of *F*^2^ > σ(*F*^2^) is used only for calculating *R*-factors(gt) *etc*. and is not relevant to the choice of reflections for refinement. *R*-factors based on *F*^2^ are statistically about twice as large as those based on *F*, and *R*- factors based on ALL data will be even larger.

### Fractional atomic coordinates and isotropic or equivalent isotropic displacement parameters (Å^2^)

**Table:**

	*x*	*y*	*z*	*U*_iso_*/*U*_eq_	
Pt1	0.14514 (4)	0.09470 (4)	0.38595 (2)	0.03996 (10)	
Br1	−0.13276 (14)	0.10500 (13)	0.27310 (7)	0.0679 (3)	
C1	0.0998 (13)	−0.1007 (10)	0.4243 (7)	0.052 (2)	
H1	0.0112	−0.1408	0.3768	0.063*	
C2	0.0164 (14)	−0.0373 (11)	0.5001 (7)	0.059 (3)	
H2	−0.1215	−0.0403	0.5006	0.071*	
C3	0.109 (2)	−0.0160 (13)	0.6061 (8)	0.091 (4)	
H3A	0.0092	−0.0039	0.6575	0.110*	
H3B	0.1742	−0.0887	0.6258	0.110*	
C4	0.2370 (16)	0.0801 (14)	0.6157 (9)	0.091 (4)	
H4A	0.3361	0.0563	0.6640	0.109*	
H4B	0.1715	0.1472	0.6474	0.109*	
C5	0.3254 (13)	0.1214 (11)	0.5168 (7)	0.066 (3)	
H5	0.3742	0.2032	0.5213	0.079*	
C6	0.4194 (13)	0.0527 (11)	0.4448 (9)	0.060 (3)	
H6	0.5218	0.0947	0.4086	0.072*	
C7	0.4495 (15)	−0.0771 (15)	0.4554 (9)	0.082 (4)	
H7A	0.4850	−0.0929	0.5279	0.098*	
H7B	0.5562	−0.0985	0.4116	0.098*	
C8	0.2914 (17)	−0.1587 (11)	0.4282 (9)	0.083 (4)	
H8A	0.2879	−0.2229	0.4795	0.099*	
H8B	0.3171	−0.1943	0.3600	0.099*	
C9	0.2351 (11)	0.2549 (9)	0.3069 (7)	0.043 (2)	
H9A	0.3702	0.2569	0.3041	0.064*	
H9B	0.1900	0.3235	0.3447	0.064*	
H9C	0.1854	0.2558	0.2365	0.064*	

### Atomic displacement parameters (Å^2^)

**Table:**

	*U*^11^	*U*^22^	*U*^33^	*U*^12^	*U*^13^	*U*^23^
Pt1	0.03947 (15)	0.03876 (19)	0.04166 (15)	−0.0015 (2)	−0.00064 (15)	0.00082 (18)
Br1	0.0593 (5)	0.0812 (9)	0.0631 (5)	0.0112 (9)	−0.0145 (5)	0.0043 (6)
C1	0.062 (6)	0.030 (5)	0.065 (6)	−0.018 (6)	−0.020 (4)	0.009 (5)
C2	0.055 (6)	0.064 (8)	0.059 (6)	−0.017 (6)	0.006 (5)	0.012 (6)
C3	0.130 (11)	0.100 (11)	0.045 (6)	0.011 (10)	0.012 (7)	0.012 (7)
C4	0.079 (7)	0.128 (14)	0.066 (7)	0.002 (10)	−0.021 (6)	−0.017 (11)
C5	0.057 (6)	0.078 (10)	0.063 (6)	−0.004 (7)	−0.025 (5)	−0.024 (6)
C6	0.035 (5)	0.059 (8)	0.086 (7)	−0.008 (5)	−0.013 (5)	0.009 (6)
C7	0.060 (6)	0.095 (12)	0.089 (8)	0.020 (9)	−0.017 (6)	−0.003 (9)
C8	0.106 (10)	0.045 (8)	0.097 (9)	0.009 (8)	−0.006 (7)	0.006 (7)
C9	0.038 (4)	0.029 (6)	0.061 (5)	−0.008 (5)	0.004 (4)	0.007 (5)

### Geometric parameters (Å, °)

**Table:**

Pt1—C5	2.118 (8)	C4—H4A	0.9700
Pt1—C6	2.138 (9)	C4—H4B	0.9700
Pt1—C9	2.151 (9)	C5—C6	1.367 (14)
Pt1—C2	2.261 (10)	C5—H5	0.9800
Pt1—C1	2.262 (11)	C6—C7	1.473 (18)
Pt1—Br1	2.4410 (11)	C6—H6	0.9800
C1—C2	1.334 (13)	C7—C8	1.488 (17)
C1—C8	1.508 (14)	C7—H7A	0.9700
C1—H1	0.9800	C7—H7B	0.9700
C2—C3	1.515 (13)	C8—H8A	0.9700
C2—H2	0.9800	C8—H8B	0.9700
C3—C4	1.415 (17)	C9—H9A	0.9600
C3—H3A	0.9700	C9—H9B	0.9600
C3—H3B	0.9700	C9—H9C	0.9600
C4—C5	1.478 (14)		
			
C5—Pt1—C6	37.5 (4)	C5—C4—H4A	108.3
C5—Pt1—C9	93.9 (4)	C3—C4—H4B	108.3
C6—Pt1—C9	94.3 (4)	C5—C4—H4B	108.3
C5—Pt1—C2	80.5 (4)	H4A—C4—H4B	107.4
C6—Pt1—C2	90.1 (4)	C6—C5—C4	126.8 (12)
C9—Pt1—C2	164.3 (4)	C6—C5—Pt1	72.1 (5)
C5—Pt1—C1	93.1 (4)	C4—C5—Pt1	111.4 (7)
C6—Pt1—C1	80.9 (4)	C6—C5—H5	113.1
C9—Pt1—C1	161.4 (4)	C4—C5—H5	113.1
C2—Pt1—C1	34.3 (3)	Pt1—C5—H5	113.1
C5—Pt1—Br1	160.4 (3)	C5—C6—C7	124.3 (11)
C6—Pt1—Br1	162.1 (3)	C5—C6—Pt1	70.4 (5)
C9—Pt1—Br1	85.8 (2)	C7—C6—Pt1	112.3 (7)
C2—Pt1—Br1	94.6 (2)	C5—C6—H6	114.0
C1—Pt1—Br1	93.3 (2)	C7—C6—H6	114.0
C2—C1—C8	127.3 (9)	Pt1—C6—H6	114.0
C2—C1—Pt1	72.8 (7)	C6—C7—C8	118.3 (10)
C8—C1—Pt1	107.1 (7)	C6—C7—H7A	107.7
C2—C1—H1	113.7	C8—C7—H7A	107.7
C8—C1—H1	113.7	C6—C7—H7B	107.7
Pt1—C1—H1	113.7	C8—C7—H7B	107.7
C1—C2—C3	122.1 (10)	H7A—C7—H7B	107.1
C1—C2—Pt1	72.9 (6)	C7—C8—C1	115.1 (10)
C3—C2—Pt1	107.0 (8)	C7—C8—H8A	108.5
C1—C2—H2	115.5	C1—C8—H8A	108.5
C3—C2—H2	115.5	C7—C8—H8B	108.5
Pt1—C2—H2	115.5	C1—C8—H8B	108.5
C4—C3—C2	118.3 (10)	H8A—C8—H8B	107.5
C4—C3—H3A	107.7	Pt1—C9—H9A	109.5
C2—C3—H3A	107.7	Pt1—C9—H9B	109.5
C4—C3—H3B	107.7	H9A—C9—H9B	109.5
C2—C3—H3B	107.7	Pt1—C9—H9C	109.5
H3A—C3—H3B	107.1	H9A—C9—H9C	109.5
C3—C4—C5	116.0 (10)	H9B—C9—H9C	109.5
C3—C4—H4A	108.3		
			
C5—Pt1—C1—C2	68.1 (6)	C9—Pt1—C5—C6	−92.0 (7)
C6—Pt1—C1—C2	103.7 (6)	C2—Pt1—C5—C6	102.8 (7)
C9—Pt1—C1—C2	−179.9 (9)	C1—Pt1—C5—C6	70.7 (7)
Br1—Pt1—C1—C2	−93.3 (5)	Br1—Pt1—C5—C6	179.4 (7)
C5—Pt1—C1—C8	−56.6 (7)	C6—Pt1—C5—C4	−123.3 (13)
C6—Pt1—C1—C8	−21.0 (7)	C9—Pt1—C5—C4	144.7 (9)
C9—Pt1—C1—C8	55.4 (13)	C2—Pt1—C5—C4	−20.6 (9)
C2—Pt1—C1—C8	−124.7 (9)	C1—Pt1—C5—C4	−52.6 (9)
Br1—Pt1—C1—C8	142.0 (7)	Br1—Pt1—C5—C4	56.1 (15)
C8—C1—C2—C3	−0.9 (18)	C4—C5—C6—C7	−0.5 (16)
Pt1—C1—C2—C3	−99.6 (10)	Pt1—C5—C6—C7	−104.2 (10)
C8—C1—C2—Pt1	98.8 (11)	C4—C5—C6—Pt1	103.7 (9)
C5—Pt1—C2—C1	−110.0 (6)	C9—Pt1—C6—C5	90.8 (7)
C6—Pt1—C2—C1	−73.7 (6)	C2—Pt1—C6—C5	−74.1 (7)
C9—Pt1—C2—C1	179.9 (11)	C1—Pt1—C6—C5	−107.3 (8)
Br1—Pt1—C2—C1	89.1 (5)	Br1—Pt1—C6—C5	−179.4 (8)
C5—Pt1—C2—C3	9.2 (8)	C5—Pt1—C6—C7	120.0 (12)
C6—Pt1—C2—C3	45.6 (8)	C9—Pt1—C6—C7	−149.1 (9)
C9—Pt1—C2—C3	−60.9 (16)	C2—Pt1—C6—C7	45.9 (9)
C1—Pt1—C2—C3	119.2 (10)	C1—Pt1—C6—C7	12.7 (9)
Br1—Pt1—C2—C3	−151.7 (8)	Br1—Pt1—C6—C7	−59.4 (15)
C1—C2—C3—C4	84.4 (16)	C5—C6—C7—C8	79.3 (14)
Pt1—C2—C3—C4	4.3 (14)	Pt1—C6—C7—C8	−1.6 (14)
C2—C3—C4—C5	−22.8 (17)	C6—C7—C8—C1	−17.9 (16)
C3—C4—C5—C6	−53.1 (15)	C2—C1—C8—C7	−54.7 (15)
C3—C4—C5—Pt1	30.0 (14)	Pt1—C1—C8—C7	26.4 (12)
